# Complete mitochondrial genome of *Dryophytes suweonensis* (Anura Hylidae)

**DOI:** 10.1080/23802359.2016.1275833

**Published:** 2017-01-05

**Authors:** Amaël Borzée, Chelsea Didinger, Yikweon Jang

**Affiliations:** a Laboratory of Behavioral Ecology and Evolution, School of Biological Sciences, Seoul National University, Seoul, Republic of Korea;; b Division of EcoScience, Ewha Womans University, Seoul, Republic of Korea

**Keywords:** *Dryophytes suweonensis*, Hylidae, mt genome

## Abstract

*Dryophytes suweonensis* is an endangered species with fragmented and declining populations from the Korean peninsula. We described 17,448 bp of *D. suweonensis* mtDNA, which had a shorter D-loop than other closely related species. The variation in nucleotide composition was similar to that of *Hyla tsinlingensis* but was larger than the one of its sister clade, *D. japonicus*.

Comparative genetic analyses between the widespread *Dryophytes japonicus* and the Endangered *D. suweonensis* are scarce, only specifying that the two species diverged about 5 mya (Li et al. 2015). Variations in behavioural ecology are now better understood, with clear behavioural and ecological differentiations between the two species (Jang et al. 2011; Borzée et al. 2016). Sequencing the full mtDNA loop of *D. suweonensis* will enable the future development of primers to help identify populations of importance for conservation.

One *D. suweonensis* was caught in Pyeongtaek, Republic of Korea (37.001°N; 127.0055°E) in June 2015 for buccal swabbing. DNA was extracted (Qiagen DNeasy, Hilden, Germany) following the instructions of the manufacturer. Primers for two long and accurate (LA) PCRs were designed based on *Hyla tsinlingensis* mtDNA (GenBank accession KP212702), with *ad hoc* primers for the D-loop due to size difference with other hylids (Geneious v. R 9; Auckland, New Zealand). Sanger sequencing was conducted by Cosmogenetech (Seoul, Republic of Korea), before GenBank upload under accession number KX854020.

The nucleotide composition for *D. suweonensis* was 29.3% of A, 27.2% of C, 15.0% of G, and 28.6% of T. The total nucleotide composition was 17,448 bp long, thus 847 bp shorter than *Hyla tsinlingensis,* with only 0.1% variation in the C nucleotide frequency (Huang et al. 2014). However, when compared with *D. japonicus* (accession number IABHU6123), it was 2071 bp shorter, with a variation in nucleotide frequency of 0.3% of A, 1.8% of C, 0.5% of G, and 1.9% of T. Using the Geneious Tree Builder plugin on Geneious v. R 9, a Neighbour-Joining Tree was constructed under the Tamura–Nei model and a 65% (5/−4) cost matrix, based on the complete mitogenetic sequences of all 11 *Hyla* uploaded on GenBank (Figure 1), and with *Bombina orientalis* as an outgroup. The grouping of *D. suweonensis* and two *H. tsinlingensis* together, while another *H. tsinlingensis* is clustered within the *Hyla* clade, indicates a potential miss-identification of the first two *H. tsinlingensis* individuals. An alternative identification would be *D. immaculatus*, due to the close relatedness with *D. suweonensis* (Li et al. 2015), and thus highlighting the divergence between these two species.

**Figure 1.**
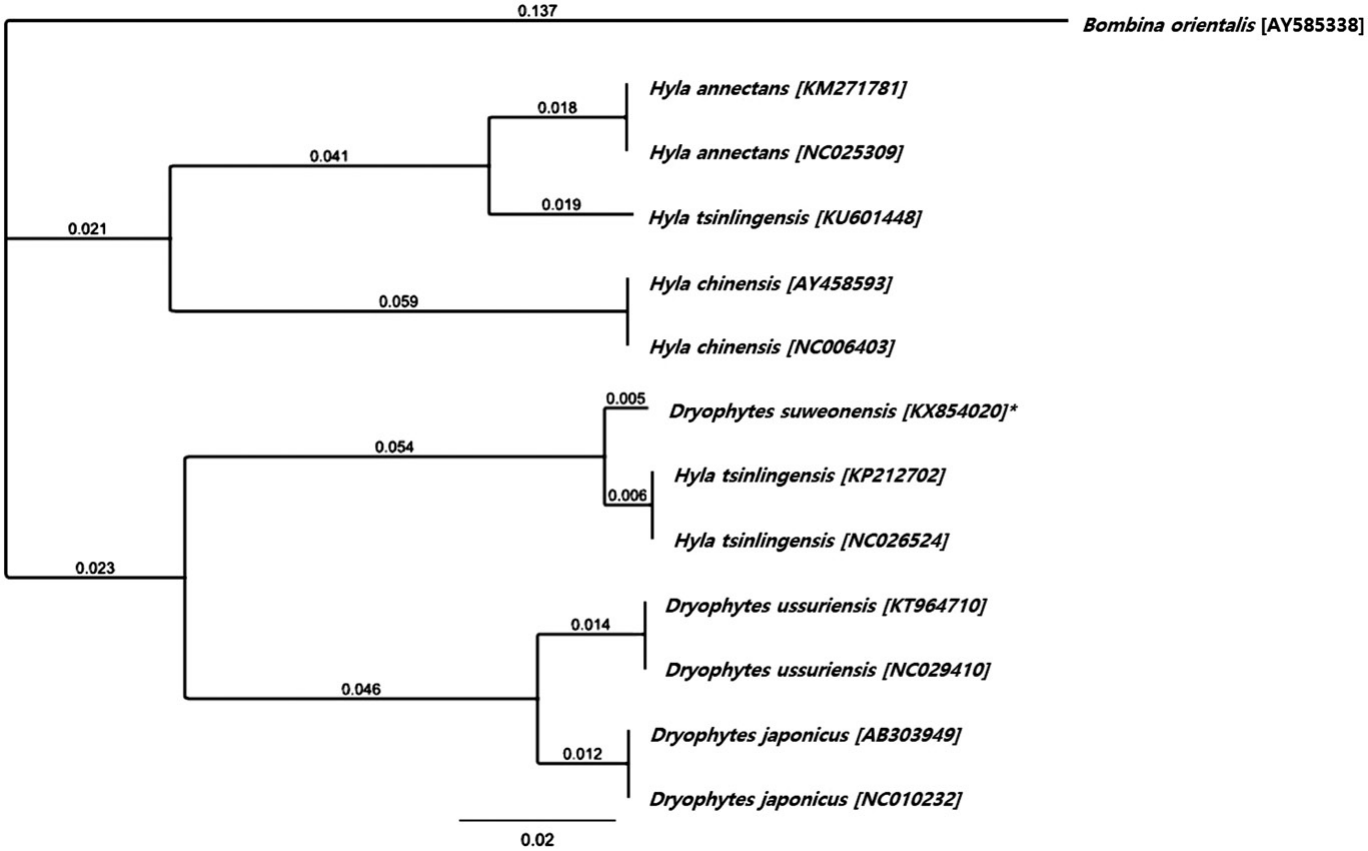
Neighbour-Joining Tree using all complete mitogenome sequences available for the sister-genera *Hyla* and *Dryophytes*, with *B. orientalis* as an outgroup. The asterisk indicate the individual sampled in this study. Branch labels are substitutions per site.
